# Prevention of sickness absence through early identification and rehabilitation of at-risk patients with musculoskeletal disorders (PREVSAM): a cost-effectiveness analysis comparing team-based rehabilitation in primary care vs treatment as usual

**DOI:** 10.1186/s12913-026-15652-2

**Published:** 2026-09-26

**Authors:** Lena Bornhöft, Annika Ekhammar, Mikael Svensson, Maria Dottori, Maria E. H. Larsson

**Affiliations:** 1 Research, Education, Development & Innovation, Primary Health Care, Region Västra Götaland, Gothenburg, Sweden; 2 School of Public Health and Community Medicine, Institute of Medicine, Sahlgrenska Academy, University of Gothenburg, Gothenburg, Sweden; 3 Department of Health and Rehabilitation, Institute of Neuroscience and Physiology, Sahlgrenska Academy, University of Gothenburg, Gothenburg, Sweden; 4 Närhälsan Primary Care Rehabilitation, Göteborg, Region Västra Götaland Sweden

**Keywords:** Cost-effectiveness, Interdisciplinary, Rehabilitation, Musculoskeletal, Sickness absence, Primary care, Secondary prevention

## Abstract

**Background:**

The PREVention of Sickness Absence through early identification and rehabilitation of at-risk patients with Musculoskeletal disorders (PREVSAM) model for team-based rehabilitation in primary care of patients with higher risk for sickness absence was investigated in a randomised controlled trial. Participants were screened with the Örebro Musculoskeletal Pain Screening Questionnaire – short form using a minimum value of 40 points for inclusion in the trial.

**Methods:**

This cost-effectiveness analysis (CEA) was performed alongside the PREVSAM trial from both healthcare and societal perspectives regarding costs in relation to change in quality-adjusted life-years (QALYs) over one year for groups receiving care according to the PREVSAM model and treatment as usual.

**Results:**

Both team-based rehabilitation and treatment as usual led to similar QALYs over one year. From the healthcare perspective, the PREVSAM model was somewhat more costly, with higher rehabilitation costs not being fully outweighed by lower costs for other primary care, specialised care, and medication. From the societal perspective, there was essentially no difference in total costs, as lower production loss costs essentially compensated for the higher rehabilitation costs. The probability that the PREVSAM model is cost-effective was close to 50% across conventional Swedish cost-effectiveness thresholds (€43 000 to €86 000 per QALY).

**Conclusions:**

The PREVSAM model and treatment as usual led to similar health outcomes and to similar total societal costs. Neither strategy was shown to be preferable on economic grounds at conventional cost-effectiveness thresholds. High individual variation implies that the screening process needs to be further refined to better identify those patients truly in need of team-based care and to facilitate appropriate resource stratification.

**Trial registration:**

The trial was registered with ClinicalTrials.gov, Protocol ID: NCT03913325. Study Registration Date, First submitted 09/04/2019, First posted 12/04/2019.

## Background

The prevalence of musculoskeletal disorders (MSDs) is immense and continually increasing globally [1, 2]. The economic burden of MSDs includes healthcare visits, investigations and treatments and extensive production loss as people of working age are often afflicted. In 2019, approximately 1.7 billion people were estimated to have MSDs in need of rehabilitation and both prevalence and years lived with disability had increased by approximately 60% over the preceding 20 years [2].

Many MSDs are short-term and benign, resolving themselves without much input from healthcare. However, a substantial proportion of MSDs are chronic or episodic health problems which affect quality of life and work ability in the long term [3]. If cases with higher risk for chronic health problems can be differentiated reliably, it is possible that healthcare resources could be directed to where they would potentially have most effect [4]. Screening tools have been developed to identify patients who run a higher risk of developing long-term pain symptoms and needing sickness absence from work because of their MSD [5]. Several studies indicate good effects of stratifying patients according to risk level [6–8].

Primary care and primary care rehabilitation are, in many countries, overburdened and subject to financing systems which do not always facilitate best evidence care [2]. Sweden has a relatively well-established primary care rehabilitation system and regulations are conducive to seeking care from rehabilitation professionals. In western Sweden, there is, however, limited economic incitement for cooperative team-based care between either medical and rehabilitation professionals or between various rehabilitation professions. Compensation schemes by the regional health authority prioritise many short patient consultations. Primary healthcare clinics and rehabilitation clinics often have separate organisations and regulations. Access to behavioural therapists is low and there is, in many cases, little contact between them and other rehabilitation professionals.

Patients with MSDs who have long-term and resource-demanding conditions likely have greater need of coordinated team-based care from several professions than do patients who recover quickly [9, 10]. However, waiting until patients develop such conditions before resources are assigned is inefficient and leads to high healthcare utilisation and much, possibly unnecessary, suffering [9]. Screening patients to determine risk for long-term pain conditions could help direct resources to those who may have greater need and help prevent development of long-term conditions [9, 11]. Based on the hypothesis that the best time to initiate coordinated team-based care would be as early as possible in the “at-risk” patient’s journey through healthcare, a model for care was developed and tested in a randomised controlled trial (RCT) [12]. According to the model, all patients with MSDs are screened for risk factors when they first seek help at rehabilitation clinics. Those who are deemed to be “at-risk” for long-term conditions and sickness absence were then randomised to care according to the PREVention of Sickness Absence for Musculoskeletal disorders (PREVSAM) model or to treatment as usual (TAU).

Team-based person-centred care may lead to higher utilisation of healthcare resources in the short term, but it is possible that this may be compensated for by a decreased need for both healthcare and sickness absence in the longer term [13, 14]. Cost-effectiveness analyses (CEAs) performed in conjunction with RCTs to investigate the relation between costs and health effects can add important knowledge to guide implementation strategies. A CEA can be made from the healthcare perspective where only healthcare costs are considered or from the societal perspective where all relevant costs for the patient, healthcare and society are included [15]. Since MSDs both require considerable healthcare resources and make a substantial contribution to sickness absence, both the healthcare and the societal perspectives are important.

The aim of this study was, therefore, to investigate the cost-effectiveness of early team-based rehabilitation of “at-risk” patients with MSDs according to the PREVSAM model from both healthcare and societal perspectives. The study is based on an RCT investigating patients who participated in early team-based rehabilitation in primary care according to the PREVSAM model and patients who had standard rehabilitation management for MSDs [16].

## Methods

### Study population and setting

The study is set in primary care rehabilitation clinics in western Sweden. There are no formal or legal limits to access physiotherapy or occupational therapy in Sweden, and no physician referrals are required. Rehabilitation is financed primarily through regional taxes and there are a multitude of both publicly- and privately-run clinics who are compensated by the regional authority for each visit. Patients pay a nominal amount per visit up to an annual limit after which visits are free.

The study population is comprised of adults seeking primary care rehabilitation for MSD. Patients who contacted one of the eight participating primary care rehabilitation clinics in western Sweden for MSD and were deemed to be at-risk for long-term health problems and needing sickness absence after screening with the Örebro Musculoskeletal Pain Screening Questionnaire – Short Form (ÖMPSQ-SF) were considered eligible [17]. At least 40 points on ÖMPSQ-SF was an inclusion criterion.

### Study design

The PREVSAM model has been described earlier [12, 13] and focuses on person-centred team-based rehabilitation in primary care. According to the model, physiotherapists and occupational therapists made their own assessments but with interdisciplinary cooperation, engaged in team meetings and, together with the patient, co-created well-defined health plans encompassing treatments, follow-ups and patient and caregiver responsibilities. The patients also had timely access to a psychotherapist and, according to patient preferences, collaboration between rehabilitation professionals and the patient’s employer. Most treatment sessions were given individually and psychotherapist sessions were given digitally.

Treatment as usual (TAU) included standard physiotherapist and/or occupational therapist treatments and strategies as deemed necessary by the individual caregiver with no particular focus on interdisciplinary collaboration and without the easily accessed psychotherapist resource.

Eligible and willing patients were randomised to either the PREVSAM model or TAU 1:1 in blocks of six. Treatment period lengths were individually determined by the responsible physiotherapist or occupational therapist. Participants were followed by the research group for one year. Treatment periods were generally considerably shorter than one year. The year-long follow-up was intended to compare the more long-term effects of the two rehabilitation strategies.

### Health outcomes

Several health outcomes were measured in the RCT at five timepoints – baseline, 1 month and 3, 6, and 12 months. Quality-adjusted life-years (QALYs) based on health-related quality of life was chosen as the outcome of greatest interest for this cost-effectiveness analysis. Health-related quality of life was measured with the Euroqol-5 dimensions-5L (EQ5D-5L) instrument [18]. The EQ5D-5L has a question-based index value which can be transformed into a point value based on population-based reference values. In this case, the Swedish EQ5D-5L value set was used [19]. Digital links to the questionnaire were sent to the participants at the five follow-up times. Quality-adjusted life-years were calculated for each participant based on the EQ5D-5L index and the appropriate time from baseline [20]. For each participant, QALYs were calculated by making a linear interpolation between each EQ5D-5L measurement point and then calculating the “area under the curve” (AUC).

### Economic costs

#### Healthcare costs

The eight participating rehabilitation clinics reported the number of visits to each profession involved in the project, team meetings, telephone calls, and contacts with employers. Data on healthcare visits to all other healthcare professions in primary care, medication and any subsequent costs within secondary care were extracted from regional healthcare databases.

**Primary care:** For the healthcare region where the majority of participants lived, register data included profession and number of visits but costs were not provided for primary care. To calculate healthcare costs, mean salary levels for appropriate healthcare professionals in primary care were obtained from the regional medical authority. Hourly costs for each profession including social fees were determined for each year in the study period. For rehabilitation professionals, consultation times were counted as 45 minutes for a first visit to physiotherapist and 30 minutes for any subsequent visits, 1 hour each for occupational therapist and psychotherapist visits, and 1 hour for team meetings. Telephone calls were counted as 15 minutes each and have been assigned to the occupational therapists as the participating rehabilitation centres did not consistently document which profession made the calls. Visits to general practitioners (GPs) and to other primary healthcare providers, such as maternal healthcare units and young adult healthcare units, were assumed to be 30 minutes, and 15 minutes were allotted to other tasks that were not patient visits. For primary care nurses, 15 minutes were counted for nurse visits and 6 minutes for other nurse tasks. A few participants belonged to another healthcare region where register data covered costs for primary care including capital and operating costs. Capital and operating costs are substantial and were included in the cost per visit for primary care in one of the two involved regions and for specialised care in both regions from the regional databases. To compensate for the discrepancy between how primary care costs were reported, costs based on number and duration of visits and salary costs were scaled up based on mean interregional hourly costs per profession to reach comparable levels to those in the region which reported costs for primary care including capital and operating costs.

**Specialised care:** Complete costs per patient including capital and operating costs were usually provided by the healthcare databases in both regions for specialised care consultations. When costs per patient were not provided, mean specialised care costs for face-to-face visits, digital visits, telephone calls and other tasks were used. Costs for radiological examinations could not be extracted from the databases but, when complete costs per person were provided for specialised care, all examination and treatment costs were included.

**Medication:** Retrieved prescriptions for medication for relevant disease categories M (Musculoskeletal system) and N (Nervous system) in the Anatomical Therapeutic Chemical (ATC) Classification System were provided by a second database. Due to integrity regulations, these could not be delivered per known participant but were anonymised before delivery and separated per group. Mean medication cost per group was added to the direct costs for each individual.

No discounting was performed as each participant was followed for only one year and salaries and other costs were extracted for the appropriate year.

#### Societal costs

The human capital approach was used in this study to value production loss [21, 22]. This approach considers all work hours lost due to sickness absence from work and relevant healthcare treatment. The Swedish Social Insurance Agency (SSIA) compensates sickness absence after the first two weeks. The number of days with compensated sickness absence was collected from the SSIA database MicroData for Analysis of Social insurance (MiDAS) between baseline and one year. Participants reported their own absence from work by weekly text message responses using the system SMS-track (SMS-Track ApS, Denmark, http://www.sms-track.com). These reports were used to capture work absence not financially compensated by SSIA and not included in the MiDAS database. Sickness absence was synchronised between the two data sources so that no day with production loss was counted twice.

Production loss due to healthcare visits was calculated based on the number of consultations to healthcare professionals and the visit lengths reported above plus one hour per visit to account for travel and waiting time for primary care visits and two hours travel and waiting time for secondary care. Days participants stayed in a hospital were counted as 24 hours per day with no added travel time.

Income level for those participants with compensated sickness absence was derived from data from the MiDAS register. Sickness absence is compensated to the individual at 80% of their registered sickness-based income. This was adjusted to reflect full-time salary and to include social fees. Participants who had no SSIA-registered sickness benefits were assigned the mean cost per time unit for those with SSIA-registered sickness absence when calculating production loss due to non-compensated sickness absence or healthcare visits.

Collection and analysis of all costing data was made in Swedish crowns (SEK) and then converted to Euros (€) at the November 2024 rate of 1 SEK = 0,086 €.

### Measures of cost-effectiveness

The incremental cost-effectiveness ratio (ICER) is the ratio between the difference between groups in mean costs and the difference between groups in mean QALYs (ΔCosts/ΔQALYs) during the one-year study period. It represents the cost to achieve one unit change in mean QALYs [15].

A cost-effectiveness plane (CE-plane) was constructed to illustrate the results. The CE-plane is divided into four quadrants (north-east, north-west, south-east, and south-west). The south-east quadrant represents the optimal results where the intervention dominates over the comparator leading to health improvements at lower costs. The north-east quadrant represents the situation where the intervention leads to health improvement but at higher cost than the comparator. In the south-west quadrant, results show that the comparator leads to more health improvement than the intervention, but the intervention has lower costs. The north-west quadrant represents the situation where the intervention is both more expensive and has worse health effects than the comparator. In the north-west quadrant, the comparator dominates over the intervention.

A cost-effectiveness acceptability curve (CEAC) is also presented. This diagram illustrates the probability that the intervention will be cost-effective for a range of cost-effectiveness thresholds (highest acceptable cost per gained QALY). There is no single official cost-effectiveness threshold in Sweden. Thresholds corresponding to SEK 500 000 (approximately €43 000) and SEK 1 000 000 (approximately €86 000) per QALY are commonly referred to for conditions of moderate and high severity, respectively, and results are therefore presented across this range [23].

Costs and QALYs were compared from both the healthcare and the societal perspective, as both perspectives give valuable knowledge about the effects of the PREVSAM model. Uncertainty from the societal perspective is assessed in the cost-effectiveness plane and cost-effectiveness acceptability curve based on 1000 bootstrapped replicates, stratified by treatment group. Differences between groups were estimated by linear regression with robust standard errors, and the analysis of QALYs was adjusted for the baseline EQ5D-5L index.

Missing EQ5D-5L index values were handled by multiple imputation using chained equations with predictive mean matching and the single nearest neighbour, generating 10 imputed datasets. The imputation model included the EQ5D-5L index at all five timepoints together with age, sex, immigration status, university-level education, baseline EQ5D VAS and baseline ÖMPSQ score. QALYs were recalculated within each imputed dataset, and estimates were combined using Rubin’s rules. Cost data were complete and were not imputed. For the cost-effectiveness plane and the acceptability curve, we drew 100 bootstrap replicates within each of the 10 imputed datasets and pooled them to 1000 replicates, so both sampling and imputation uncertainty are represented.

The study is reported in accordance with the Consolidated Health Economic Evaluation Reporting Standards 2022 (CHEERS) checklist [24].

## Results

The RCT included 253 patients from eight rehabilitation clinics, both publicly- and privately-run, who sought care between May 2019 and June 2022. A total of 128 participants were included in the PREVSAM intervention group and 125 in the TAU group. Descriptive characteristics have been published earlier as have details on the content of the intervention [13]. Participants were between 20 and 67 years of age and approximately 68% were women. There were no significant differences between groups for socioeconomic characteristics or baseline health except for occupation. Significantly more participants in the PREVSAM group described working as managers or having an occupation requiring advanced level of higher education. However, no differences were found regarding education or income level between the groups. Four participants, one from PREVSAM and three from TAU, did not agree to retrieval of sickness absence data from SSIA. One participant who was included in the 3-month evaluation [13] was later found to be ineligible for the study due to long-term full-time disability pension and was excluded from this study according to recruitment criteria.

In addition to treatment as usual, the PREVSAM model included team meetings, a structured health plan, timely access to psychotherapist and the possibility of workplace contact. Team meetings were initiated at least once for 86% of participants in the PREVSAM group and 3% in TAU, while 40% of PREVSAM participants utilised the project psychotherapist resource which was not available to the TAU group [25].

Healthcare utilisation is shown in Table 1 and costs for healthcare and production loss in Tables 2 and 3. Costs for radiological examinations are included in the costs per person provided by the healthcare registers when they were ordered by caregivers within specialised care but could not be retrieved for examinations ordered by caregivers within primary care.

**Table 1:** Healthcare utilisation for the PREVSAM and TAU groups during study period

Caregiver		**Consultations***(Total, mean (SD))*			
Care level/contact type	Profession	PREVSAM (n = 128)	TAU (n = 125)	∆ (PREVSAM-TAU)	P
*Primary care rehabilitation visits*	PT	885 6.9 (6.4)	756 6.1 (6.6)	+0.8	0.291
	OT	200 1.6 (1.8)	87 0.7 (1.9)	**+0.9**	**<0.001**
	Psychologist	243 1.9 (2.4)	0 0 (0)	**+1.9**	**<0.001**
	Team (PT+OT)	188 1.5 (0.9)	6 0.1 (0.3)	**+1.4**	**<0.001**
*Primary care rehabilitation telephone contacts*		148 1.2 (1.4)	79 0.6 (1.2)	**+0.6**	**0.001**
*Primary care visits*	Physician	347 2.7 (2.4)	335 2.7 (2.6)	0	0.920
	Nurse	115 0.9 (1.4)	114 0.9 (1.6)	0	0.944
	Other	22 0.2 (0.8)	12 0.1 (0.7)	+0.1	0.525
*Primary care telephone and other contacts*	Physician	362 2.8 (3.4)	430 3.4 (3.9)	−0.6	0.183
	Nurse	1078 8.4 (12.9)	886 7.1 (12.1)	+0.7	0.397
	Other	5 0.0 (0.3)	1 0.0 (0.1)	0	0.414
*Specialised care visits*	Various	224 1.8 (2.8)	318 2.5 (5.5)	−0.7	0.148
*Specialised care telephone and other contacts*	Various	39 0.3 (0.8)	80 0.6 (1.7)	−0.3	0.051
*Specialised care hospital stays (days)*	Various	22 0.2 (1.0)	42 0.3 (2.1)	−0.1	0.423
**Other aspects for care period**					
*Medication*	Prescriptions	447 3.5 (5.5)	789 6.3 (14.1)	**−2.8**	**0.039**
	DDD for ATC-groups M and N	22 125.7 172.9 (321.4)	47 585.3 380.7 (2250.1)	−207.8	0.308
*Sickness absence (days)*	SSIA-compensated	2970.5 23.2 (55.0)	3974.75 31.8 (78.0)	−8.6	0.314
	Other self-reported	1735 13.6 (23.7)	1477 11.8 (20.8)	+1.8	0.536

PREVSAM = intervention group; TAU = treatment as usual group; PT = physiotherapist; OT = occupational therapist; DDD = Defined Daily Doses; ATC = Anatomical Therapeutic Chemical Classification System-groups *M* = Musculoskeletal system and *N* = Nervous system; SSIA = Swedish Social Insurance Agency. Significant differences shown in boldface

**Table 2:** Mean direct costs per person for the PREVSAM and TAU groups during study period

Caregiver		**Direct costs (€)***(Mean (95% CI))*			
Care level	Profession/contact type	PREVSAM (n = 128)	TAU (n = 125)	∆ Mean costs (PREVSAM-TAU)	P
*Primary care rehabilitation*	PT	654 (555;754)	575 (471;678)	+79.66 (−63.08;222.39)	0.273
	OT	342 (284;400)	156 (95;217)	**+186.14** **(102.29;269.98)**	**<0.001**
	Psychologist	440 (343;585)	0 (0;0)	**+439.90** **(343.26;536.54)**	**<0.001**
	Team (PT+OT)	530 (476;585)	17 (−1;35)	**+513.37** **(456.50;570.24)**	**<0.001**
	**Total rehab**	1 967 (1758;2 176)	748 (621;875)	**+1 219** **(975.82;1 462.31)**	**<0.001**
*Primary care*	Physician	916 (786;1 046)	994 (837;1 151)	−77.54 (−280.48;125.40)	0.452
	Nurse	206 (158;254)	181 (134;227)	+25.69 (−41.01;92.38)	0.449
	Other	19 (4;35)	12 (0;25)	+7.22 (−12.86;27.31)	0.479
	**Total primary care**	1 142 (987;1 297)	1 193 (1 004;1 381)	−50.88 (−293.85;192.08)	0.680
*Secondary care*	Out-patient	654 (482;826)	1 180 (740;1 620)	**-526.24 (−997.72;-54.77)**	**0.029**
	Hospital stays	318 (25;612)	403 (28;778)	−84.45 (−558.84;389.94)	0.726
	**Total secondary care**	973 (616;1 329)	1 583 (934;2 232)	−610.69 (−1 348.61;127.22)	0.104
**Total mean healthcare consultation costs per person**		4 081 (3 596;4 567)	3 536 (2 752;4 320)	+545.19 (−373.13;1 463.52)	0.243
**Other direct costs**					
Medication*	Cost per person	37 (24;50)	83 (37;128)	−45.37 (−92.67;1.93)	0.060

€=Euro; CI = confidence interval; PREVSAM = intervention group; TAU = treatment as usual group; PT = physiotherapist; OT = occupational therapist; *For ATC-groups M (musculoskeletal system) and N (nervous system), medication data separated by group but not connected to known specific individuals; Conversion Swedish crowns to Euro was made at 1 SEK = 0.086 €. Significant differences shown in boldface

**Table 3:** Mean production loss costs for the PREVSAM and TAU groups during study period

Grounds for production loss		**Production loss costs (€)***(Mean (95% CI))*			
	Details	PREVSAM (n = 128)	TAU (n = 125)	∆ Mean costs (PREVSAM-TAU)	P
*Healthcare consultations*	Including travel and waiting time	481 (399;563)	432 (294;570)	+49.44 (−110.67;209.55)	0.543
*SSIA-compensated sickness absence*	Register data	2 738 (1 581;3 895)	3 642 (2 040;5 244)	−904.20 (−2 871.30;1 062.90)	0.366
*Other sickness absence*	Self-reported data	1 589 (1 094;2 084)	1 297 (921;1 674)	+291.51 (−327.92;910.95)	0.355
**Total production loss costs**		4 808 (3 432;6 183)	5 371 (3 664;7 078)	−563.25 (−2 745.23;1 618.74)	0.612

€=Euro; CI = confidence interval; PREVSAM = intervention group; TAU = treatment as usual group; SSIA = Swedish Social Insurance Agency; Conversion Swedish crowns to Euro was made at 1 SEK = 0.086 €

Health-related quality of life improved in both groups over the study year. The mean EQ5D-5L index increased from 0.73 (95% CI 0.69;0.76) at baseline to 0.85 (0.81;0.89) at 52 weeks in the PREVSAM group and from 0.72 (0.68;0.76) to 0.86 (0.82;0.90) in the TAU group. The mean improvement from baseline to 52 weeks was 0.126 (95% CI 0.079;0.173) in the PREVSAM group and 0.134 (0.082;0.187) in the TAU group, both *p* < 0.001, with no difference between groups in the magnitude of improvement (−0.008, 95% CI −0.077;0.060, *p* = 0.81).

The complete case analysis, which includes participants with one-year values for EQ5D and their costs, is presented in Table 4 from the healthcare perspective and societal perspectives. The multiple imputation analysis from the societal perspective is presented in Table 5. Both groups accumulated approximately 0.84 QALYs over the study year, with small and statistically non-significant differences between groups in both the complete case and the multiple imputation analyses.

**Table 4:** Complete case analysis (*n* = 159) from healthcare and societal perspectives: mean incremental costs and QALYs

	PREVSAM *(95% CI)n* = 88	TAU *(95% CI)n* = 71	∆ (PREVSAM-TAU)
Mean primary care rehabilitation costs *(€)*	2 115 (1 858; 2 372)	808 (613;1 003)	**+1306.82** **(986.21;1627.44)**
Mean primary care costs *(€)*	1 150 (962;1 338)	1 139 (911;1 367)	+11.14 (−281.75;304.04)
Mean secondary care costs *(€)*	922 (482;1 363)	1 554 (816;2 291)	−631.42 (−1484.98;222.14)
Mean medication costs *(€)*	38 (38;38)	82 (82;82)	**−44.52** **(−44.52;-44.52)**
Mean total healthcare costs *(€)*	4 224 (3 642;4 808)	3 583 (2 676;4 490)	641.86 (−426.58;1 710.29)
Mean production loss costs *(€)*	5 488 (3 631;7 346)	5 417 (3 287;7 548)	71.05 (−2 731.69;2 873.79)
Mean total costs *(€)*	9 713 (7 673;11,753)	9 000 (6 336;11 665)	712.91 (−2 613.67;4 039.49)
Mean QALYs	0.84 (0.81;0.87)	0.85 (0.83;0.88)	−0.01 (−0.05;0.03)

PREVSAM = treatment according to PREVSAM model; TAU = treatment as usual; ∆=difference; €=euros; QALYs = quality-adjusted life-years; CI = confidence intervals; QALY differences are adjusted for the baseline EQ5D-5L index. Significant differences shown in boldface

**Table 5:** Multiple imputation analysis (*n* = 253) from the societal perspective: mean incremental costs, QALYs

	PREVSAM (95% CI)	TAU (95% CI)	∆ (PREVSAM-TAU) (95% CI)
Mean total healthcare costs *(€)*	4 126 (3 634;4 617)	3 600 (2 821;4 378)	526.01 (−390.12;1 442.13)
Mean production loss costs *(€)*	4,808 (3 432;6 183)	5,371 (3 664;7 078)	−563.25 (−2 744.68;1 618.18)
Mean total costs *(€)*	8,933 (7 353;10 514)	8,970 (6 722;11 219)	−37.24 (−2 772.15;2 697.66)
QALYs	0.84 (0.81;0.87)	0.84 (0.81;0.87)	0.00 (−0.03;0.04)

PREVSAM = treatment according to PREVSAM model; TAU = treatment as usual; ∆=difference; €=euros; QALYs = quality-adjusted life-years; CI = confidence intervals; QALY differences are adjusted for the baseline EQ5D-5L index

There were limited missing cost data. Sickness absence register data was complete except for the four participants who did not agree to retrieval of sickness absence data from the MiDAS register. These four participants had in total 10 days in the PREVSAM group and 34 days in the TAU group for self-reported sickness absence. Self-reported sickness absence had considerable missing (16% PREVSAM, 21% TAU) [16] but was used only for periods not covered by the MiDAS register. More of the self-reported sickness absence was missing for the TAU group which could potentially lead to an underestimation of the costs for production loss for TAU.

**Fig. 1:**
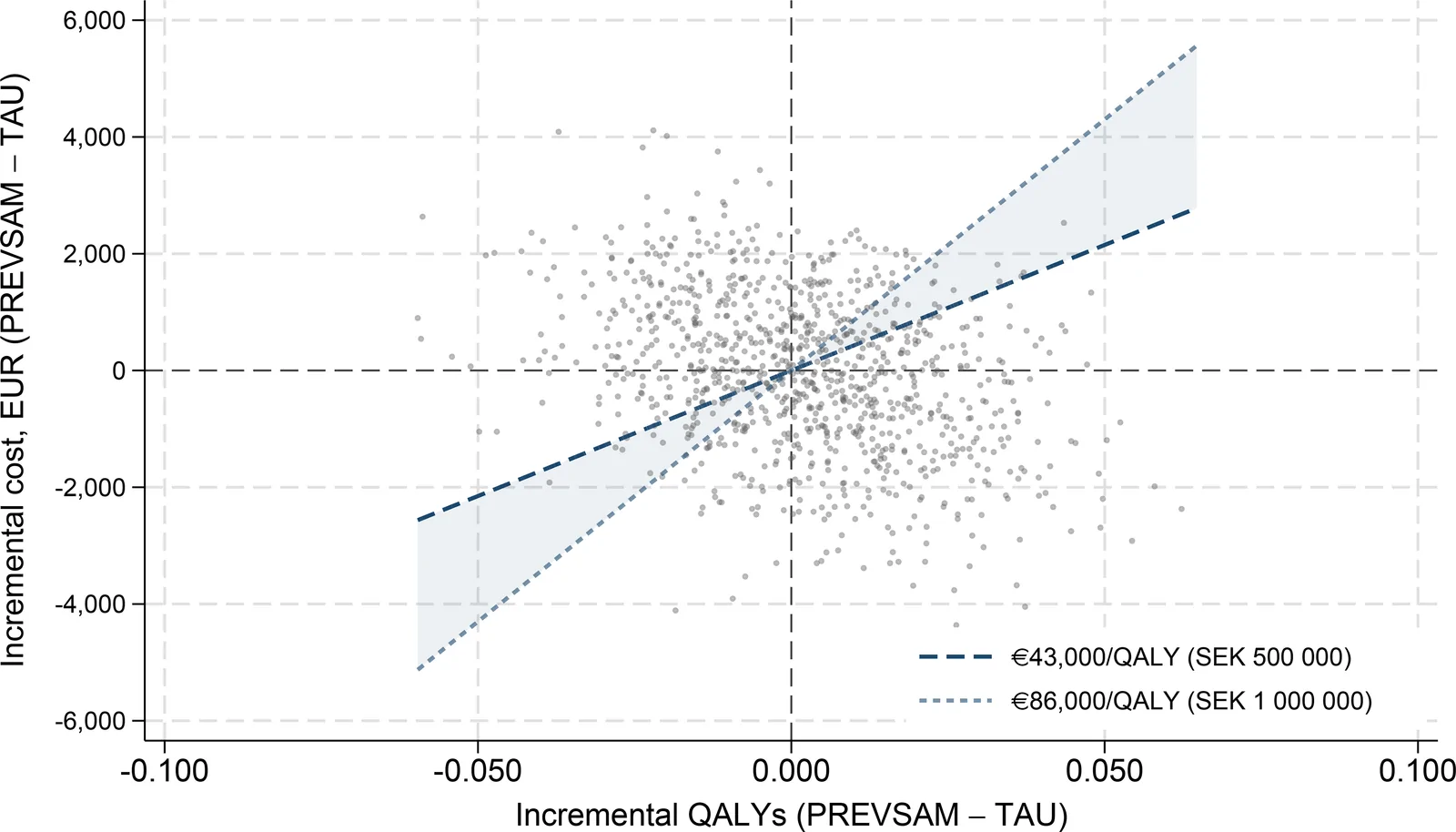
Cost-effectiveness plane, societal perspective, multiple imputation analysis (*n* = 253). Each point represents one of 1000 bootstrapped replicates of the incremental cost and incremental QALY difference (PREVSAM minus TAU). The shaded cone spans cost-effectiveness thresholds from €43 000 per QALY (SEK 500 000) to €86 000 per QALY (SEK 1 000 000)

Because the difference between groups in mean QALYs was close to zero, the denominator of the incremental cost-effectiveness ratio approaches zero. The ratio is therefore not meaningfully defined and is not reported. Uncertainty from the societal perspective is illustrated in the cost-effectiveness plane (Fig. 1), based on 1000 bootstrapped replicates of the multiple imputation analysis. Of these replicates, 31.5% fell in the south-east quadrant (PREVSAM less costly and more effective) and 28.7% in the north-west quadrant (more costly and less effective), while 50.2% were cost-saving and 52.6% showed a gain in QALYs.

**Fig. 2:**
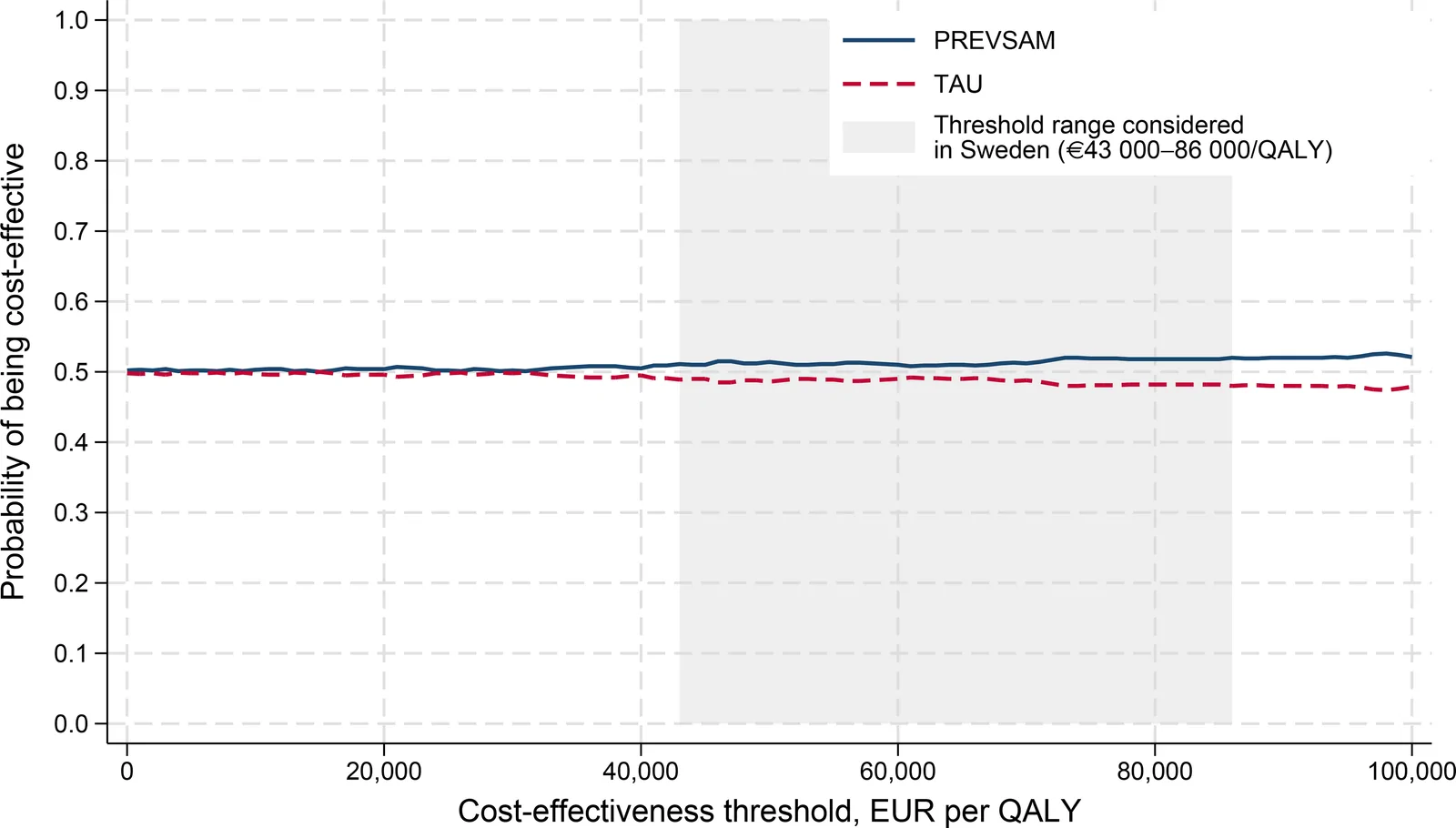
Cost-effectiveness acceptability curve, societal perspective, multiple imputation analysis (*n* = 253). Probability that each strategy is the more cost-effective option at each threshold, based on the same 1000 bootstrapped replicates. The shaded band marks the €43 000 to €86 000 per QALY range

The cost-effectiveness acceptability curve (Fig. 2) shows that the probability that the PREVSAM model is cost-effective remains close to 50% across the whole range of cost-effectiveness thresholds (51.5% at €43 000 and 51.3% at €86 000 per QALY). The curve is nearly horizontal because the difference between groups in mean QALYs is close to zero.

## Discussion

This cost-effectiveness analysis compared the health effects and costs of team-based rehabilitation of patients with musculoskeletal disorders who were deemed to be “at risk” for developing long-term health problems and sickness absence with treatment as usual of this patient group from both a healthcare perspective and a societal perspective. The CEA indicates minimal differences between groups both in health effects and in total societal costs, and approximately an equal probability that either strategy is the more cost-effective. While team-based rehabilitation resulted in higher costs for rehabilitation professionals, it was partially compensated by lower costs for primary care physicians, for specialised care, for sickness absence and for medication. Both team-based rehabilitation and treatment as usual led to improved health-related quality of life between baseline and one year but with minimal difference between groups.

Effects of team-based rehabilitation and treatment as usual on several health outcomes have been analysed earlier [16]. Both management strategies were associated with considerable health improvement indicating that both are relevant clinical choices for the patient group investigated here.

The two perspectives point in different directions. From the healthcare perspective the PREVSAM model was somewhat more costly (+€526, 95% CI −390;1 442) without a corresponding gain in QALYs. From the societal perspective the difference in total costs was essentially nil (-€37, 95% CI −2 772;2 698), because the higher rehabilitation costs were offset by lower costs for other primary care, specialised care, medication and, in particular, production loss (-€563).

In a study of costs of production loss and primary care interventions, it was shown that rehabilitation activity for MSD accounted for only 3.7% of the costs generated by production loss [26]. In the present study, two active rehabilitation strategies were compared, both showing a low entry to the sickness benefit system. These findings may suggest that continued investment in rehabilitation is warranted as societal costs for MSD are driven primarily by production losses.

It is possible that the difference between groups for production loss due to sickness absence is underestimated due to more missing in the TAU group for self-reported sickness absence. Another possibility is, according to experiences from healthcare professionals and patients who participated in the team-based rehabilitation, that some patients benefited greatly from it and others little or not at all [27, 28]. The lack of difference between the groups in health-related quality of life may be due to the large variation in individual patients’ needs. Qualitative studies and a process evaluation indicate that certain participants may have received all treatment and support that they required from a single caregiver, while others may have found other benefits than those measured with EQ5D-5L [25, 27, 28]. Perhaps more refined screening with other cut-offs on the screening instrument or more information to participants about team-based care before inclusion may improve early identification of patients in need of team-based care.

### Specialised care

The lower costs for specialised care were unexpected. Patients in the intervention group had considerably more visits with rehabilitation professionals as was expected. Patients in both groups consulted their primary care clinics at comparable levels but it seems that fewer were referred to specialised care. It is possible that the team-based rehabilitation increased patients’ confidence in the primary care management. It is also possible that the more extensive medical records that were likely available in conjunction with team-based rehabilitation increased the confidence among other caregivers within primary care that the health condition was being managed acceptably within the primary care setting. Both could reduce the need for assessment and treatment within secondary care. Similarly, experiences within diabetes care have shown that teamwork within primary care is associated with reduced need for specialised care [29].

### Medication

The number of prescriptions and costs for medication were also lower in the PREVSAM group. A recent extensive literature-based analysis suggests that both physiotherapy and team-based support may have positive effect on reducing consumption of pain medication [30]. Alternative methods for pain management may have been offered to a greater extent in the PREVSAM group leading to a lower demand for pain medication.

### Study limitations

When RCTs are conducted pragmatically within real-life healthcare settings with many participants and caregivers involved, certain difficulties arise. Many different caregivers are giving person-centred care to many patients with a wide range of health problems. The randomisation process succeeded in a relatively good distribution of demographic factors between the two groups but data on health problems was not collected or analysed for impact.

Missing values for all repeated measures led to increased uncertainty. As the health effects were so similar between groups, a few missing values can tip the scale between being able to show a positive effect of an intervention or not. Costing results could be affected by the higher level of missing values for self-reported sickness absence in the TAU group. Also, there is a ceiling for sickness absence compensation from the SSIA. It is unknown how many of the participants with compensated sickness absence had salaries higher that the compensation ceiling which can lead to an underestimation of sickness absence costs in both groups. However, there was no significant difference in income between groups so it is unlikely that any such underestimation would change the overall results.

Cost data were complete for all 253 participants, whereas the complete case analysis in Table 4 is restricted to the 159 participants who also had a one-year QALY. Participants excluded from the PREVSAM group had considerably lower total costs than those included (€7 218 versus €9 713), while excluded and included participants in the TAU group had similar costs (€8 932 versus €9 000). Restricting the analysis to complete cases therefore shifts the difference between groups in total costs from -€37 in the imputed analysis to +€713. The multiple imputation analysis, which retains all randomised participants, can therefore be seen as the more appropriate basis for inference and the complete case analysis could more be seen as a sensitivity analysis.

In sum, this CEA adds to the collective understanding of the effects of a complicated intervention in a real-life primary care setting and can help stakeholders decide on resource distribution.

## Conclusion

Over one year, team-based rehabilitation according to the PREVSAM model and treatment as usual led to similar improvements in health-related quality of life for those patients with MSD screened to have at least moderate risk for developing long-term health problems and needing sickness absence, and to similar total societal costs. From the healthcare perspective the PREVSAM model was somewhat more costly without a corresponding gain in QALYs, whereas from the societal perspective the higher rehabilitation costs were offset by lower costs for other care and for sickness absence. At conventional cost-effectiveness thresholds in Sweden, the probability of PREVSAM being cost-effective was close to 50%, so neither strategy was shown to be preferable on economic grounds. High individual variation implies that the screening process needs to be further refined to better identify those patients truly in need of team-based care and to facilitate appropriate resource stratification.

## Data Availability

The datasets used and analysed in the current study are available from the corresponding author on reasonable request.

## Abbreviations

ATC: Anatomical Therapeutic Chemical Classification System including categories M (Musculoskeletal system) and N (Nervous system)
AUC: Area under the curve
CE-plane: Cost-effectiveness plane
CEA: Cost-effectiveness analysis
CEAC: Cost-effectiveness acceptability curve
CI: Confidence interval
EQ5D-5L: Euroqol 5 dimensions, 5 levels
€: Euro
GP: General practitioner
ICER: Incremental cost-effectiveness ratio
MiDAS: MicroData for Analysis of Social insurance
MSD: Musculoskeletal disorder
PREVSAM: PREVention of Sickness Absence for Musculoskeletal disorders
QALY: Quality-adjusted life-years
RCT: Randomised controlled trial
SEK: Swedish crowns
SSIA: Swedish Social Insurance Agency
TAU: Treatment as usual
ÖMPSQ-SF: Örebro Musculoskeletal Pain Screening Questionnaire – short form
